# Real-world use of first-line pembrolizumab + platinum + taxane combination regimens in recurrent / metastatic head and neck squamous cell carcinoma

**DOI:** 10.3389/fonc.2024.1348045

**Published:** 2024-02-08

**Authors:** Christopher M. Black, Dandan Zheng, Gleicy M. Hair, Lei Ai, Liya Wang, Daisuke Goto, Nati Lerman, Behzad Bidadi, Glenn J. Hanna

**Affiliations:** ^1^ Center for Observational and Real-World Evidence (CORE), Merck & Co., Inc., Rahway, NJ, United States; ^2^ Oncology Late Stage Development, Merck & Co., Inc., Rahway, NJ, United States; ^3^ Center for Head & Neck Oncology, Dana-Farber Cancer Institute, Boston, MA, United States

**Keywords:** head and neck squamous cell carcinoma, antineoplastic agents, PD-1, taxanes, Kaplan-Meier estimate, patient outcomes, real-world observational study, treatment patterns

## Abstract

**Introduction:**

The programmed death-1 (PD-1) immune checkpoint inhibitor pembrolizumab is currently approved in the US for the first-line (1L) treatment of recurrent or metastatic head and neck squamous cell carcinoma (R/M HNSCC), either alone or in combination with platinum and 5-fluorouracil (5-FU). However, the toxicity of 5-FU has motivated the study of alternate combinations that replace 5-FU with a taxane. The objective of the current study was to describe the baseline characteristics, treatment patterns and sequences, and real-world outcomes of individuals receiving pembrolizumab + platinum + taxane as 1L treatment for R/M HNSCC in the US.

**Methods:**

This was a retrospective study of US adults ≥18 years of age receiving pembrolizumab + platinum + taxane as 1L treatment for R/M HNSCC, using electronic health record data from a nationwide de-identified database. Real-world overall survival (rwOS), time on treatment (rwToT), and time to next treatment (rwTTNT) outcomes were assessed using Kaplan–Meier analysis.

**Results:**

The study population comprised 83 individuals (80.7% male) with a median age of 64 years. The most common tumor site was the oropharynx (48.2%); 70.0% of these tumors were HPV-positive. A total of 71.1% of the study population had an Eastern Cooperative Oncology Group performance status of 0–1 at index date, 71.8% had a combined positive score for programmed death ligand-1 (PD-L1) expression of ≥1, and 30.8% had a score of ≥20. The median (95% CI) rwOS was 14.9 (8.8–23.3) months, rwToT was 5.3 (4.0–8.2) months, and rwTTNT was 8.7 (6.8–12.3) months. Among the 24 individuals who received a subsequent therapy, the most common second-line therapies were cetuximab-based (*n* = 9) or pembrolizumab-containing (*n* = 8) regimens.

**Conclusions:**

The rwOS and other real-world outcomes observed for this study population further support pembrolizumab + platinum + taxane combination therapy as a potential 1L treatment option for R/M HNSCC.

## 1 Introduction

Head and neck squamous cell carcinoma (HNSCC) is a common cancer in the US, especially among men (1–4). Risk factors include tobacco and alcohol use and infection with oncogenic strains of human papillomavirus (HPV) (2, 4). The treatment of recurrent or metastatic disease (R/M HNSCC) is challenging. The median overall survival (OS) on the previous standard first-line (1L) therapy for R/M HNSCC, namely cetuximab + platinum + 5-fluorouracil (5-FU), was less than 1 year (5, 6).

In 2019, the US Food and Drug Administration (FDA) approved the programmed death-1 (PD-1) immune checkpoint inhibitor pembrolizumab (KEYTRUDA^®^) for 1L treatment of R/M HNSCC, either as monotherapy or in combination with platinum and 5-FU (7). In the registration clinical trial (KEYNOTE-048), pembrolizumab + platinum + 5-FU was associated with longer OS compared with cetuximab + platinum + 5-FU in all individuals (median OS: 13.0 versus 10.7 months), while pembrolizumab monotherapy was associated with longer OS compared with cetuximab + platinum + 5-FU in individuals with a combined positive score (CPS, a measure of programmed death ligand-1 [PD-L1] expression in tumor and infiltrating immune cells) of ≥1 (12.3 versus 10.3 months in the CPS ≥1 group and 14.9 versus 10.7 months for the CPS ≥20 group) (8). Both pembrolizumab-containing therapies were better tolerated than the previous standard of care (8). In a previous study investigating the real-world post-approval use of pembrolizumab, we found that the real-world OS (rwOS) outcomes of individuals receiving pembrolizumab monotherapy or pembrolizumab + platinum + 5-FU were numerically similar to the OS outcomes of the corresponding groups in the KEYNOTE-048 trial (9, 10). The 2024 NCCN^1^ Clinical Practice Guidelines in Oncology (NCCN Guidelines^®^) for Head and Neck Cancers list pembrolizumab + platinum (cisplatin or carboplatin) + 5-FU as a category 1 option for 1L treatment of R/M HNSCC, with pembrolizumab monotherapy as a category 1 option for tumors that express PD-L1 with CPS ≥1 (11).

In our previous real-world study, we found that 103 (13.8%) individuals receiving a 1L pembrolizumab-containing therapy for R/M HNSCC did not receive an FDA-approved regimen (9, 10). The most common alternate regimens were pembrolizumab + carboplatin + paclitaxel (*n* = 52) and pembrolizumab + carboplatin (*n* = 13). These findings may be related to 5-FU’s toxicity (particularly with respect to gastrointestinal side-effects) and patient inconvenience [i.e., the need for port placement and a 96-hour pump to administer the drug, and a requirement for dihydropyrimidine dehydrogenase deficiency testing before initiating treatment (12)]. Taxanes such as paclitaxel have been suggested as replacements for 5-FU in both pembrolizumab and cetuximab combination therapies for R/M HNSCC and have demonstrated encouraging performance in initial analyses (12–19). Pembrolizumab in combination with cisplatin or carboplatin plus paclitaxel or docetaxel is currently listed as a category 2A option for 1L treatment of R/M HNSCC in the NCCN Guidelines^®^ (11), and thus is already available in the US. In initial results from the single-arm Phase 4 KEYNOTE-B10 trial of pembrolizumab + carboplatin + paclitaxel as 1L treatment for R/M HNSCC, this combination therapy demonstrated antitumor activity and had a manageable safety profile that was consistent with the known safety profiles of each constituent therapy (16, 20).

The primary objective of the current study was to describe the baseline demographic and clinical characteristics and the rwOS, real-world time on treatment (rwToT), and real-world time to next treatment (rwTTNT) outcomes of individuals receiving pembrolizumab + platinum + taxane combination therapy as 1L treatment for R/M HNSCC in real-world US settings. We also aimed to describe the second-line (2L) treatments received by this study population.

## 2 Methods

### 2.1 Study design

This was a retrospective observational study using deidentified electronic health record data. The index date was defined as the date of initiation of 1L pembrolizumab + platinum + taxane combination therapy within the cohort selection period of July 1, 2019 to June 30, 2022. The baseline period was defined as the period between each individual’s first database record (with the earliest possible date being the database inception date of January 1, 2011) and their index date. The follow-up period of ≥6 months ran from the index date until the last observed visit, record of death, or data cut-off on December 31, 2022, whichever occurred first.

### 2.2 Study population

The study population comprised individuals with electronic health record data in the Flatiron Health advanced HNSCC database (21–23). The database includes structured and unstructured longitudinal, patient-level data from ~280 cancer clinics and ~800 sites of care. All data are deidentified in accordance with the US Health Insurance Portability and Accountability Act, and no specific institutional review board approvals or informed consent were required (24). Eligible individuals were ≥18 years of age at the time of diagnosis of advanced HNSCC in the hypopharynx, larynx, oropharynx, or oral cavity, selected using International Classification of Diseases (ICD) codes 140x, 141x, 143x, 144x, 145x, 146x, 147x, 148x, 149x, or 161x (ICD-9)/C00x, C01x, C02x, C03x, C04x, C05x, C06x, C09x, C10x, C11x, C12x, C13x, C14x, or C32x (ICD-10). Advanced disease (R/M HNSCC) was defined as Stage IVC HNSCC or HPV-positive Stage IV oropharyngeal cancer, HNSCC not cured at initial diagnosis^2^, HNSCC not cured at first locoregional recurrence, HNSCC with second locoregional recurrence, or HNSCC with distant recurrence. All diagnoses were confirmed via manual chart review. Eligible individuals initiated pembrolizumab + platinum + taxane combination therapy as 1L treatment for R/M HNSCC during the cohort selection period. Exclusion criteria were HNSCC at any tumor site not listed above, a record of any platinum-containing therapy ≤6 months prior to the index date, or receipt of a clinical trial drug at any time during the study period.

### 2.3 Study measures

Baseline clinical characteristics were summarized using all available information from the baseline period, while baseline demographic characteristics were summarized using the closest available record from before or ≤30 days after the index date.

### 2.4 Study outcomes

First-line regimens were defined using all eligible agents for which there was a record in the database on or within 28 days after the index date. Treatment discontinuation was defined as initiation of the next line of therapy (LOT), record of death while receiving the LOT, or a gap of ≥120 days between last therapy administration date and last follow-up date. Substitution between platinum therapy agents with no other changes to the treatment regimen or seamless transitions from pembrolizumab + platinum + taxane therapy to pembrolizumab monotherapy without disease progression (for example as maintenance therapy) did not advance the LOT. Second-line regimens were defined using all agents for which there was a record in the database after 1L treatment discontinuation. All lines of therapy in the study database were rules-based and defined by oncology expert clinicians.

Real-world time on treatment was defined as the interval in months between 1L treatment initiation and 1L treatment discontinuation, including any treatment interruptions or breaks of ≤90 consecutive days in length; real-world time to next treatment as the interval in months between the index date and the start of the next LOT; and rwOS as the interval in months between the index date and the date of death from any cause.

### 2.5 Statistical analysis

Study measures and outcomes were summarized using descriptive statistics (medians, ranges, interquartile ranges [IQRs], and CIs for continuous variables and percentages for categorical variables). Kaplan–Meier curves were generated to report rwOS, rwToT, and rwTTNT outcomes. No hypothesis testing or formal tests of difference were conducted.

## 3 Results

### 3.1 Study population

We included 83 eligible individuals who received 1L pembrolizumab + platinum + taxane. The median (IQR) age of the study population was 64 (57–72) years and a majority (80.7%) of the group were male (**Table 1**). Around three-quarters (75.9%) had a history of smoking. The most common tumor sites were the oropharynx (48.2%) and larynx (25.3%); 70.0% of oropharyngeal tumors were HPV-positive. Fifty-nine individuals (71.1%) had an Eastern Cooperative Oncology Group performance status (ECOG PS) of 0–1 at index date, 14 (16.9%) had an ECOG PS of 2–4, and this information was missing for 10 individuals (12.0%). Evidence of PD-L1 expression testing was present for 57 individuals (68.7%), and specifically CPS testing for 39 (47.0%). Among individuals with a record of a CPS test, 5 (12.8%) had a CPS <1, 28 (71.8%) had a CPS ≥1, 12 (30.8%) had a CPS ≥20, and the CPS was not documented for 6 individuals (15.4%).

**Table 1:** Baseline demographic and clinical characteristics of individuals receiving 1L pembrolizumab + platinum + taxane combination therapy.

Characteristic	1L pembrolizumab + platinum + taxane (*N* = 83)
Age (years)	
Median (IQR)	64 (57–72)
Sex	
Male	67 (80.7)
Smoking status	
History of smoking	63 (75.9)
Practice type	
Community	54 (65.1)
Academic	24 (28.9)
Both	5 (6.0)
Primary tumor site(s)	
Oropharynx	40 (48.2)
Oral cavity	16 (19.3)
Larynx	21 (25.3)
Hypopharynx	6 (7.2)
HPV status (oropharynx subtype only) ^A^	(*N* = 40)
Positive	28 (70.0)
Advanced diagnostic criteria	
HNSCC with distant recurrence	31 (37.3)
Stage IVC or HPV-positive Stage IV oropharyngeal tumor at diagnosis	21 (25.3)
HNSCC with locoregional recurrence ^B^	16 (19.3)
HNSCC not cured at initial diagnosis ^C^	15 (18.1)
ECOG PS on index date	
0–1	59 (71.1)
2–4	14 (16.9)
Unknown/not documented	10 (12.0)
Evidence of PD-L1 testing ^D^	
Yes	57 (68.7)
CPS ^E^	(*N* = 39)
<1	5 (12.8)
≥1	28 (71.8)
≥20	12 (30.8)
Unknown/not documented	6 (15.4)

1L, first-line; CPS, combined positive score; ECOG PS, Eastern Cooperative Oncology Group performance status; HNSCC, head and neck squamous cell carcinoma; HPV, human papilloma virus; IQR, interquartile range; PD-L1, programmed death ligand 1. All values are presented as n (%) unless otherwise indicated.

^A^Status determined based on HPV testing, p16 testing, or both.

^B^Includes individuals not cured at first locoregional recurrence, or with second locoregional recurrence.

^C^In the study database, individuals are categorized as not cured at initial diagnosis if they did not have Stage IVC or HPV-positive Stage IV oropharyngeal tumor at diagnosis and meet any of the following 3 criteria: 1) did not receive any therapy to treat the primary tumor; 2) received only chemotherapy to treat the primary tumor (i.e., no surgery or radiation); 3) had progressive disease during initial therapy and/or persistent disease following initial therapy of the primary tumor.

^D^PD-L1 testing performed at index date ± 30 days.

^E^If multiple CPS values were available, the score recorded closest to the index date was reported.

### 3.2 First-line treatment patterns

The most common platinum and taxane therapies were carboplatin and paclitaxel, respectively. Carboplatin was received by 80 individuals, with a median (IQR) cycle number of 5 (3–7) and a median (IQR) first dose (among *n* = 77 individuals with available dosage data) of 400 mg (246–596 mg). The median (IQR) number of cycles among the 75 individuals who received paclitaxel was 6.0 (4–8), and the median (IQR) first dose was 238 mg (146–337 mg; *n* = 71). Eight individuals received docetaxel (median [IQR] 4 [3–6] cycles, 123 mg [83–148 mg] first dose) and 3 received cisplatin (median [IQR] 6 (4–8) cycles, 122 mg [121–126 mg] first dose).

### 3.3 Real-world outcomes

**Figure 1:**
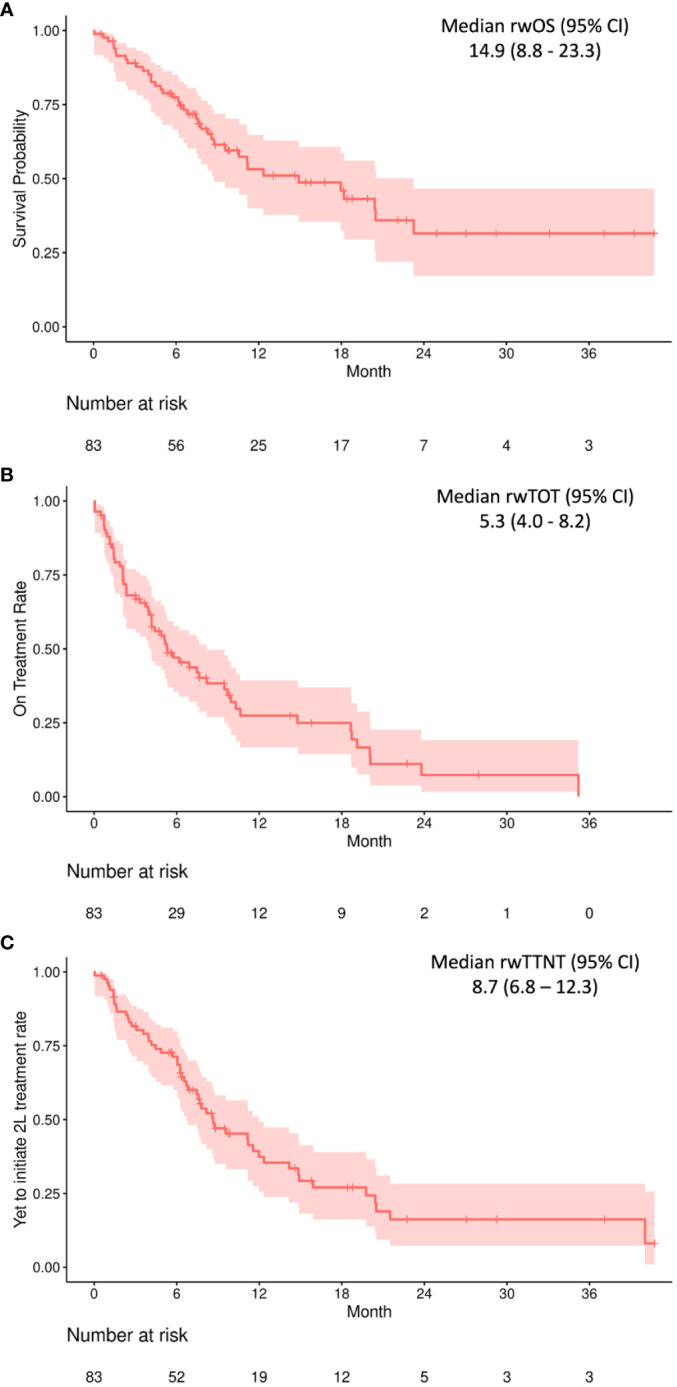
Real-world overall survival **(A)**, real-world time on treatment **(B)**, and real-world time to next treatment **(C)** outcomes among 83 individuals receiving 1L pembrolizumab + platinum + taxane combination therapy. CI, confidence interval. All values provided in months.

Real-world outcomes are presented in months in **Table 2** and as Kaplan–Meier curves in **Figure 1**. The median (IQR) follow-up period was 7.7 (5.2–15.2) months. The median (95% CI) rwOS was 14.9 (8.8–23.3) months, with an OS rate of 53.2% at 12 months and 31.4% at 24 months. The median (95% CI) rwTOT was 5.3 (4.0–8.2) months; 27.4% of the study population were on treatment at 12 months and 7.4% at 24 months. The median (95% CI) rwTTNT was 8.7 (6.8–12.3) months.

**Table 2:** Real-world overall survival, time on treatment, and time to next treatment among individuals receiving 1L pembrolizumab + platinum + taxane combination therapy.

Outcome	1L pembrolizumab + platinum + taxane (*N* = 83)
Follow-up (months)	
Median (IQR)	7.7 (5.2–15.2)
Median (range)	7.7 (0.0–40.7)
rwOS (months)	
Median (95% CI)	14.9 (8.8–23.3)
OS rate: % (95% CI)	
At 6 months	77.4 (66.6–85.1)
At 12 months	53.2 (40.0–64.7)
At 18 months	46.0 (32.5–58.5)
At 24 months	31.4 (17.3–46.6)
rwToT (months)	
Median (95% CI)	5.3 (4.0–8.2)
On treatment rate: % (95% CI)	
At 6 months	47.0 (35.4–57.8)
At 12 months	27.4 (16.6–39.3)
At 18 months	24.9 (14.4–37.0)
At 24 months	7.4 (1.7–19.1)
rwTTNT (months)	
Median (95% CI)	8.7 (6.8–12.3)
Yet to initiate 2L treatment: % (95% CI)	
At 6 months	71.3 (60.0–79.9)
At 12 months	37.4 (25.5–49.2)
At 18 months	27.0 (16.2–39.0)
At 24 months	16.2 (7.3–28.3)

1L, first-line; 2L, second-line; CI, confidence interval; IQR, interquartile range; OS, overall survival; rwOS, real-world overall survival; rwToT, real-world time on treatment; rwTTNT, real-world time to next treatment.

### 3.4 Subsequent therapy

Twenty-nine individuals (34.9%) died during or after 1L treatment and 24 (28.9%) moved to 2L therapy (**Table 3**; follow-up period as above). The most common 2L agents were cetuximab-based (*n* = 9) and pembrolizumab-containing (*n* = 8) regimens; the latter group included 2 individuals receiving pembrolizumab monotherapy, 1 receiving a pembrolizumab + platinum + 5-FU regimen, and 3 individuals re-treated with pembrolizumab + platinum + taxane.

**Table 3:** Subsequent therapies among individuals receiving 1L pembrolizumab + platinum + taxane combination therapy.

Outcome	1L pembrolizumab + platinum + taxane (*N* = 83)
Died after 1L treatment	29 (34.9)
Moved to 2L therapy	24 (28.9)
Cetuximab-based regimens ^A^	9 (10.8)
Pembrolizumab monotherapy	2 (2.4)
Pembrolizumab + platinum + 5-FU	1 (1.2)
Other pembrolizumab-containing therapy ^B^	5 (6.0)
Nivolumab	2 (2.4)
Platinum-based chemotherapy	1 (1.2)
Non-platinum chemotherapy	2 (2.4)
Other ^C^	2 (2.4)
Censored without 2L therapy at cut-off date	30 (36.1)

1L, first-line; 2L, second-line; 5-FU, 5-fluorouracil. All values are presented as n (%) unless otherwise indicated.

^A^Cetuximab monotherapy (n = 7); carboplatin, cetuximab, docetaxel; carboplatin, cetuximab, paclitaxel.

^B^Capecitabine, cisplatin, docetaxel, pembrolizumab; carboplatin, docetaxel, pembrolizumab; carboplatin, paclitaxel protein-bound, pembrolizumab; carboplatin, paclitaxel, pembrolizumab; fluorouracil, pembrolizumab.

^C^Pembrolizumab, bicalutamide, enzalutamide, and triptorelin; cyclophosphamide, doxorubicin, rituximab-pvvr, rituximab/hyaluronidase, vincristine. These regimens were grouped in the ‘Other’ category because one or more drug component is not approved for this indication.

**Supplementary Table 1** shows subsequent therapies stratified by diagnosis. Four individuals received a 2L therapy for HNSCC not cured at initial diagnosis, 7 for HNSCC with distant recurrence, and 5 for HNSCC with locoregional recurrence. None of the group with distant recurrence received a pembrolizumab-containing therapy. A third-line treatment was documented for 3 individuals, of whom 1 received a cetuximab-containing regimen and 1 received a regimen including both cetuximab and pembrolizumab; the sole individual receiving a 4L therapy was treated with methotrexate.

## 4 Discussion

To our knowledge, this was the first study to describe the baseline characteristics and real-world outcomes of individuals receiving pembrolizumab + platinum + taxane combination therapy as 1L treatment for R/M HNSCC in real-world US settings. In a previous study, we used the same source database and methods to characterize real-world outcomes among individuals receiving pembrolizumab monotherapy or 1L pembrolizumab + platinum + 5-FU (9, 10). All real-world outcomes from the current study were numerically longer than those from the 1L pembrolizumab + platinum + 5-FU group in the prior study: rwOS, median (95% CI) 14.9 (8.8–23.3) months in the current study compared to 11.9 (9.0–16.0) months in the prior study; rwToT, 5.3 (4.0–8.2) months compared to 4.9 (3.8–5.6) months; and rwTTNT, 8.7 (6.8–12.3) months compared to 6.6 (5.8–8.3) months. The numerically longer rwToT and rwTTNT observed in the current study may in part reflect differences between 5-FU- and taxane-containing combinations in terms of tolerability (e.g., gastrointestinal side-effects) and/or patient convenience [e.g., the need for a port and 96-hour pump to administer 5-FU (12)]. A recent clinical trial comparing cetuximab-containing regimens that included platinum and 5-FU versus cisplatin and docetaxel found that individuals treated with the taxane-containing regimen had similar overall and progression-free survival compared to those receiving the 5-FU-containing, but fewer serious adverse events and better self-reported quality of life (17). Other studies have confirmed that cetuximab + cisplatin + docetaxel is a well-tolerated regimen (18, 19). Further research, including the final results of the KEYNOTE-B10 clinical trial, is needed to determine whether combining pembrolizumab with taxanes is also associated with reduced toxicity compared to 5-FU-containing combinations.

The median age and the proportions of males, tumor sites, individuals with CPS values ≥1 and ≥20, and individuals with metastatic disease in the current study were similar to those of the participants in the ongoing Phase 4 KEYNOTE-B10 trial of pembrolizumab + carboplatin + paclitaxel as 1L treatment for R/M HNSCC (16, 20). An interim analysis of the clinical trial data indicated a median OS of 12.1 months, which is consistent with the median rwOS of 14.9 months in the current study (25). However, only individuals with an ECOG PS of 0–1 were eligible for the clinical trial, whereas 16.9% of the current study population had an ECOG PS of ≥2, indicating a higher individual burden of illness (26). In addition, the current study included 15 individuals who were categorized in the study database as not cured at initial diagnosis or as having persistent disease, categories that were excluded from the KEYNOTE-B10 trial. This group included a numerically lower proportion of individuals with HPV-positive tumors and higher proportion of individuals with an ECOG status of 2–4 than did the full study population, but had otherwise similar characteristics (**Supplementary Table 2**; please note that no tests of statistical difference were conducted). Excluding these 15 individuals from the outcomes analyses did not significantly affect the results (**Supplementary Figure 1**). The current study also included a higher proportion of oropharyngeal tumors that were HPV-positive than did the clinical trial population (70.0% versus 47.6%). This difference is likely to affect OS and response to treatment, since HPV-positive tumors are generally associated with a better prognosis (16, 27–29). We were not able to stratify the outcomes analyses by HPV status due to the small sample size; further research with a larger study population would be needed to address this potential confounder. The current real-world analysis thus provides information on early indicators of regimen effectiveness and tolerability in a more clinically representative group of patients, a valuable complement to the emerging clinical trial data.

We note that our findings are not meant to provide conclusive evidence on the comparative effectiveness and tolerability of different regimens, and that we were unable to perform formal statistical tests of differences between the outcomes observed in the current and prior studies because of differences in study population characteristics and unrecorded confounding factors. Another study limitation is that data on toxicity, adverse events, and reasons for treatment selection and discontinuation are not captured in the study database, and thus we were not able to directly assess treatment tolerability or other factors that may have affected rwToT or rwTTNT. Further, CPS values, which may influence treatment selection (9, 10, 30), were not available for over half of the study population. Testing for PD-L1 prior to treatment selection is not included in the NCCN recommendation for the regimen of interest, and our findings in the current study are in line with those of a previous analysis in which we observed that 73.8% of individuals receiving 1L pembrolizumab monotherapy (for which PD-L1 testing is indicated on the NCCN label) and 67.4% of those receiving 1L pembrolizumab + chemotherapy had evidence of PD-L1 testing (9). For oropharyngeal cancers, HPV status can also affect the choice and effectiveness of immunotherapy and chemotherapy regimens (31). The data for the current study were retrospectively collected and did not allow us to fully adjust for these potential confounders. In addition, some of the estimated rwTOT outcomes reported in this study may differ from the true values because treatment discontinuation dates were not directly reported in the database but rather defined by a rule using proxy dates (see Section 2.4), which may not reflect the actual dates on which treatments were discontinued. However, previous studies have reported that real-world outcomes data are generally consistent with the findings of randomized clinical trials, thus supporting the use of real-world data (32–35). A strength of the study is that we used data from one of the largest national longitudinal oncology database, and the study population had similar baseline characteristics to those described in previous real-world observational studies of the use of R/M HNSCC therapies that include pembrolizumab or other immunotherapies (9, 10, 36).

In conclusion, the rwOS, rwToT, and rwTTNT for 1L pembrolizumab + platinum + taxane observed in this real-world outcome analysis provide support for this regimen as a potential 1L combination therapy option for R/M HNSCC.

## Data availability statement

The original contributions presented in the study are included in the article/**Supplementary Material**. Further inquiries can be directed to Flatiron Health.

## Ethics statement

Ethical approval was not required for the study because this was a retrospective observational study using deidentified electronic health record data. The study was conducted in accordance with the local legislation and institutional requirements. Written informed consent for participation was not required from the participants or the participants’ legal guardians/next of kin in accordance with the national legislation and institutional requirements because this was a retrospective observational study using deidentified electronic health record data.

## Author contributions

CB: Conceptualization, Investigation, Writing – original draft, Writing – review & editing. DZ: Conceptualization, Investigation, Writing – original draft, Writing – review & editing. GMH: Conceptualization, Formal analysis, Investigation, Writing – original draft, Writing – review & editing. LA: Conceptualization, Formal analysis, Investigation, Writing – original draft, Writing – review & editing. LW: Conceptualization, Investigation, Writing – original draft, Writing – review & editing. DG: Conceptualization, Formal analysis, Investigation, Writing – review & editing. NL: Investigation, Writing – original draft, Writing – review & editing. BB: Investigation, Writing – original draft, Writing – review & editing. GJH: Conceptualization, Investigation, Writing – original draft, Writing – review & editing.

## Footnotes

1 NCCN=National Comprehensive Cancer Network^®^ (NCCN^®^).

2 In the study database, individuals are categorized as not cured at initial diagnosis if they did not have Stage IVC or HPV-positive Stage IV oropharyngeal tumor at diagnosis and meet any of the following 3 criteria: 1) did not receive any therapy to treat the primary tumor; 2) received only chemotherapy to treat the primary tumor (i.e., no surgery or radiation); 3) had progressive disease during initial therapy and/or persistent disease following initial therapy of the primary tumor.

## Supplementary material

Supplementary Table 1 Subsequent therapies among individuals receiving 1L pembrolizumab + platinum + taxane combination therapy, stratified by diagnosis.

Supplementary Table 2 Baseline demographic and clinical characteristics of individuals receiving 1L pembrolizumab + platinum + taxane combination therapy for head and neck squamous cell carcinoma not cured at initial diagnosis.

Supplementary Figure 1 Real-world overall survival (A), real-world time on treatment (B), and real-world time to next treatment (C) outcomes among 68 individuals receiving 1L pembrolizumab + platinum + taxane combination therapy after excluding those not cured at initial diagnosis.
