# Supplementary material for: Real-world use of first-line pembrolizumab + platinum + taxane combination regimens in recurrent / metastatic head and neck squamous cell carcinoma
Source: Front Oncol. 2024 Feb 8;14:1348045. doi: 10.3389/fonc.2024.1348045 (PMC10881782; doi:10.3389/fonc.2024.1348045)
Supplement: Supplementary Table 1 — Subsequent therapies among individuals receiving 1L pembrolizumab + platinum + taxane combination therapy, stratified by diagnosis. [file DataSheet_1.docx]

Supplementary Table 1. Subsequent therapies among individuals receiving 1L pembrolizumab + platinum + taxane combination therapy, stratified by diagnosis

| Line of therapy | Diagnosis | Therapy | *n* |
| --- | --- | --- | --- |
| 2 | HNSCC not cured at initial diagnosis | Bicalutamide, enzalutamide, pembrolizumab, triptorelin | 1 |
|  |  | Carboplatin, fluorouracil, pembrolizumab | 1 |
|  |  | Carboplatin, paclitaxel protein-bound, pembrolizumab | 1 |
|  |  | Carboplatin, paclitaxel, pembrolizumab | 1 |
|  | HNSCC with distant recurrence | Capecitabine | 1 |
|  |  | Carboplatin, cetuximab, paclitaxel | 1 |
|  |  | Cetuximab | 1 |
|  |  | Cetuximab, methotrexate | 1 |
|  |  | Fluorouracil, leucovorin, pembrolizumab | 1 |
|  |  | Nivolumab | 2 |
|  | HNSCC with locoregional recurrence | Cetuximab | 4 |
|  |  | Pembrolizumab | 1 |
| 3 | HNSCC not cured at initial diagnosis | Cetuximab, paclitaxel protein-bound, pembrolizumab | 1 |
|  | HNSCC with distant recurrence | Carboplatin, cetuximab, fluorouracil | 1 |
|  | HNSCC with locoregional recurrence | Carboplatin, paclitaxel | 1 |
| 4 | HNSCC with distant recurrence | Methotrexate | 1 |

HNSCC, head and neck squamous cell carcinoma.

Supplementary Table 2. Baseline demographic and clinical characteristics of individuals receiving 1L pembrolizumab + platinum + taxane combination therapy for head and neck squamous cell carcinoma not cured at initial diagnosis

| Characteristic | *N* = 15 |
| --- | --- |
| Age (years) |  |
| Median (IQR) | 63 (57–72) |
| Sex |  |
| Male | 12 (80.0) |
| Smoking status |  |
| History of smoking | 11 (73.3) |
| Practice type |  |
| Community | 8 (53.3) |
| Academic | 7 (46.7) |
| Primary tumor site(s) |  |
| Oropharynx | 5 (33.3) |
| Oral cavity | 5 (33.3) |
| Larynx | 4 (26.7) |
| Hypopharynx | 1 (6.7) |
| HPV status (oropharynx subtype only) ^A^ | (*N* = 10) |
| Positive | 2 (13.3) |
| ECOG PS on index date |  |
| 0–1 | 8 (53.3) |
| 2–4 | 5 (33.3) |
| Unknown / not documented | 2 (13.3) |
| Evidence of PD-L1 testing ^B^ |  |
| Yes | 11 (73.3) |
| CPS ^C^ | (*N* = 6) |
| <1 | 1 (16.7) |
| ≥1 | 3 (50.0) |
| ≥20 | 1 (16.7) |
| Unknown / not documented | 1 (16.7) |

1L, first-line; CPS, combined positive score; ECOG PS, Eastern Cooperative Oncology Group performance status; HNSCC, head and neck squamous cell carcinoma; HPV, human papilloma virus; IQR, interquartile range; PD-L1, programmed death ligand 1. All values are presented as *n* (%) unless otherwise indicated.

^A^ Status determined based on HPV testing, p16 testing, or both.

^B^ PD-L1 testing performed at index date ± 30 days.

^C^ If multiple CPS values were available, the score recorded closest to the index date was reported.

Supplementary Figure 1. Real-world overall survival (A), real-world time on treatment (B), and real-world time to next treatment (C) outcomes among 68 individuals receiving 1L pembrolizumab + platinum + taxane combination therapy after excluding those not cured at initial diagnosis

CI, confidence interval. All values provided in months.
